# Boron seed coating combined with seed inoculation with boron tolerant bacteria (*Bacillus* sp. MN-54) and maize stalk biochar improved growth and productivity of maize (*Zea mays* L.) on saline soil

**DOI:** 10.1016/j.heliyon.2023.e22075

**Published:** 2023-11-04

**Authors:** Hafiz Saqib Hayat, Atique-ur Rehman, Shahid Farooq, Muhammad Naveed, Hayssam M. Ali, Mubshar Hussain

**Affiliations:** a Department of Agronomy, Bahauddin Zakariya University, Multan, 60800, Pakistan; b Department of Plant Protection, Faculty of Agriculture, Harran University, Sanlıurfa, 63050, Turkey; c Institute of Soil and Environmental Sciences, University of Agriculture, Faisalabad, 37000, Pakistan; d Department of Botany and Microbiology, College of Science, King Saud University, P.O. Box 11451, Riyadh, Saudi Arabia; e School of Veterinary and Life Sciences, Murdoch University, 90 South Street, Murdoch, WA 6150, Australia

**Keywords:** Saline environments, Stomatal conductance, Photosynthesis, Grain protein content, Plant growth promoting bacteria

## Abstract

Salinity exerts significant negative impacts on growth and productivity of crop plants and numerous management practices are used to improve crop performance under saline environments. Micronutrients, plant growth promoting bacteria and biochar are known to improve crop productivity under stressful environments. Maize (*Zea mays* L.) is an important cereal crop and its productivity is adversely impacted by salinity. Although boron (B) application, seed inoculation with boron-tolerant bacteria (BTB) and biochar are known to improve maize growth under stressful environments, there is less information on their combined impact in enhancing maize productivity on saline soils. This study investigated the impact of B seed coating combined with seed inoculation with BTB + biochar on maize productivity under saline soil. Four B seed coating levels [0.0 (no seed coating), 1.0, 1.5, 2.0 g B kg^−1^ seed], and individual or combined application of 5 % (w/w) maize stalk biochar, and seed inoculation with *Bacillus* sp. MN-54 BTB were included in the study. Different growth and yield attributes and grain quality were significantly improved by seed coating with 1.5 B kg^−1^ seed coupled with biochar + BTB. Seed coating with 1.5 B kg^−1^ seed combined with biochar + BTB improved stomatal conductance by 32 %, photosynthetic rate by 15 %, and transpiration ratio by 52 % compared to seed coating (0 B kg^−1^ seed) combined with biochar only. Similarly, the highest plant height (189 cm), number of grain rows cob^−1^ (15.5), grain yield (54.9 g plant^−1^), biological yield (95.5 g plant^−1^), and harvest index (57.6 %) were noted for B seed coating (1.5 g B kg^−1^ seed) combined with biochar + BTB inoculation. The same treatment resulted in the highest grain protein and B contents. It is concluded that B seed coating at 1.5 g B kg^−1^ seed combined with biochar + BTB inoculation could significantly improve yield and quality of maize crop on saline soils. However, further field experiments investigating the underlying mechanisms are needed to reach concrete conclusions and large-scale recommendations.

## 1 Introduction

Saline soils are characterized by a significant accumulation of soluble salts [1], which reduce agricultural productivity by restricting plant growth [2,3]. Projections indicate that 50 % of the global lands will be impacted by salinity till 2050 [2]. Elevated salt levels in saline soils negatively impact soil structure and impede water infiltration. Furthermore, higher salt concentration significantly inhibits the activities of microorganisms necessary for nutrient cycling and breakdown of organic matter [4,5]. Elevated salt concentration exerts negative effects on the hydrological characteristics of the soil, thereby hindering the plants’ ability to acquire sufficient moisture necessary for their normal physiological functions and development [6]. Similarly, high salt concentration reduces the uptake of crucial nutrients required for plant growth. Hence, plant growth is significantly retarded by salt stress, leading to low biomass accumulation and stunted development [7]. Stunted root growth and reduced leaf under high soil salinity result in reduced crop productivity [8]. Rapidly increasing global population necessitates the utilization of saline soils in achieving future food security [9]. However, sustainable, and eco-friendly management practices are needed to improve plant growth on saline soils [9]. The use of salt-tolerant plant varieties [10], appropriate irrigation management [11], soil amendments [8], growth regulators [12], osmoprotectants [13], plant growth promoting bacteria [14,15], and micronutrients [16], could be utilized to lower the adverse impacts of soil salinity on plant growth.

Maize (*Zea mays* L.) is an important cereal crop with significant nutritional value. It contains 4.5 % fats, 71.8 % vitamins, and 10.4 % protein [17]. Grain maize is cultivated on ∼197 million hectares globally, making it the most extensively cultivated crop after wheat [18]. Maize serves as a crucial primary resource for various industries such as food, textiles, paper, and feed [19,20]. It is essential raise maize production by 67 % to meet the projected food requirements of the expanding population till 2050 [21]. Although maize is moderately salt-tolerant, salinity is a significant stress which lowers its productivity [22]. Therefore, agronomic and genetic interventions are needed to improve maize growth and productivity on saline soils.

Boron (B) is an essential micronutrient for optimum growth and productivity of agronomic crops [23]. Low availability of micronutrients and organic matter content in saline soils distributed in arid and semi-arid regions significantly reduce crop yields [24]. Higher pH, calcareous soils, insufficient organic matter, higher salt concentration, extended periods of drought, and inappropriate application of fertilizers are the primary factors responsible for B-deficiency [25]. Boron deficiency causes pollen sterility leading to reduced crop yields [26]; therefore, continuous supply of B is necessary to growing tissues and roots for maintaining optimal plant development and cell wall biosynthesis. Maize has relatively low B requirement; however, field studies identified that B-deficiency decreased maize yield in several geographic regions of the world [27]. Several studies have indicated that maize yield was significantly improved by B application [28,29]. Boron is supplied through soil and foliar applications, and seed treatments. Seed coating is an effective strategy for decreasing the adverse effects of B-deficiency on plant growth and crop yields [[30], [31], [32]]. Different adhesive materials are used to adhere the desired nutrients to the seed surface in seed coating [30]. Seed coating with micronutrients is a common technique used for the enhancement of crop performance in saline soils [33]. Seedlings require continuous supply of nutrients during initial growth stage; therefore, seed coating serves the purpose [34]. Boron seed coating significantly improved yield and grain biofortification of chickpea [32], and rice [31]. Boron has a narrow toxicity and deficiency range; therefore, optimizing seed coating is necessary before its use. However, B seed coating level have not been optimized for maize crop.

Biochar (charcoal like substance) is generated by subjecting organic biomass materials, including wood chips, crop residues, or organic waste, to pyrolysis or thermal decomposition in an environment with restricted oxygen supply [35]. Biochar application has been reported to improve plant growth on saline soils. Biochar enhances plant growth and yield through two main mechanisms on saline soils. Firstly, it increases the availability of essential nutrients such as phosphorous, potassium, calcium, and magnesium in soil. Secondly, it enhances the physical, chemical, and biological properties of soil, including pH, cation exchange capacity [[36], [37], [38], [39]]. Biochar application has been reported to improve nutrient availability to maize plants [39]. Furthermore, improved soil fertility and nutrient availability have been reported as result of biochar application [40]. Biochar improves fertilizer use efficiency and utilization of nutrients [41]. Furthermore, biochar enhances the growth of soil microorganisms [42]. Biochar provides a suitable habitat for soil microorganisms [43] through enhancing living place of microbes in saline soils [44]. Microorganisms enhance plant growth and productivity through several processes, including genetic modification, nutrient solubilization, and production of siderophores [45]. Nutrients can be moved closer to the rhizosphere of plants with the inoculation of microbes, which leads to improvement of plant growth in saline and sodic-saline soils [46].

Plant growth-promoting rhizobacteria can colonize the rhizosphere of numerous plant species and exert significant positive impacts on their growth and productivity [47]. The introduction of these bacteria to plants results in biochemical and morphological alterations that enhance their ability to withstand abiotic stresses, including salinity [48]. Bacteria exhibiting higher tolerance to salinity can significantly promote plant growth under high soil salinity [49]. *Bacillus* sp. are highly tolerant to salinity and B and significantly improve growth and productivity of several crops under saline conditions. Seed coating with B-tolerant bacteria (BTB), i.e., *Bacillus* sp. can promote growth and yield of crop plants [50]. Seed inoculation with *Bacillus* sp. MN-54 improved nutrient availability and yield of maize crop [51].

Although the individual effects of B and biochar application, and seed inoculation with BTB on growth and productivity of maize are known, no study has investigated their interactive effects on growth and productivity of maize crop on saline soil. Therefore, this study investigated the interactive effect of B seed coating, seed inoculation with BTB and biochar application on water relations, photosynthesis, growth, productivity, and grain quality of maize crop grown on saline soil. It was hypothesized that interactive effect of B seed coating, biochar application and seed inoculation with BTB will increase growth and yield of maize under saline conditions.

## 2 Materials and methods

### 2.1 Experimental site

**Fig. 1:**
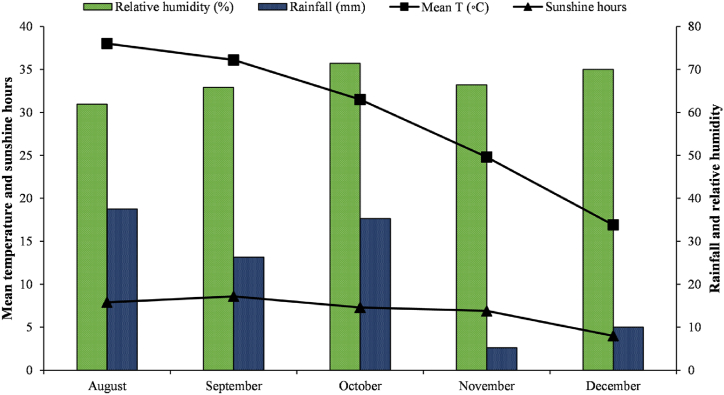
Weather data of the experimental site during the crop growth period. The values are monthly averages. (Source: Central Cotton Research Institute (CCRI), Multan, Pakistan).

This study was conducted at Agronomic Research Area, Bahauddin Zakariya University, Multan (30.27° N, 71.50° E, at 129 m elevation), Pakistan under semi-arid conditions. Experimental soil was collected from a nearby salt-affected field (30.260611° N, 71.514771° E) and analyzed prior to experiment. The soil was sandy-loam with 8.5 pH, 0.47 % soil organic matter, 4.3 dSm^−1^ electrical conductivity, 5.7 mg kg^−1^ available phosphorus, 118 mg kg^−1^ available potassium and 33 % soil saturation. The soil was B-deficient (0.43 mg kg^−1^). The experiment was done under glasshouse conditions. The weather data of the experimental site during the study are presented in Fig. 1. The mean temperature and sunshine hours decreased from August to December. Relative humidity ranged 60–70 % during the experimental duration.

### 2.2 Plant materials and treatments

Seeds of hybrid maize cultivar ‘DK-67142’ were purchased from Monsanto Seeds Private Limited Pakistan and used in the study. Biochar was prepared by dry distillation of maize stalks at 500 °C. Biochar had 398.37 mg kg^−1^ organic carbon content, 8.56 pH, 20.45 cmol kg^−1^ cation exchange capacity, 49.28 % carbon content, 0.98 % nitrogen content, 0.21 % phosphorus 0.24 % boron, 0.8 % calcium and cadmium in trace amounts. Biochar has the potential to store carbon in the soil for longer periods as compared to unparalyzed organic material [52].

Bacterial strain *Bacillus* sp. MN-54 was collected from the Soil and Environmental Microbiology Laboratory, Institute of Soil and Environmental Science, University of Agriculture, Faisalabad, Pakistan (GenBank accession number KT375574). This strain has been used for enhancing the growth and productivity of various crops [53]. *Bacillus* sp. MN-54 (10 %) was cultured in 250-mL Erlenmeyer flask containing tryptic soy broth medium at 28 ± 2 °C and 180 rpm (Firstek, Scientific, Tokyo Japan) for 72 h. Before seed inoculation, the optical density of culture was measured at 600 nm using a spectrophotometer (Thermo Electron Corporation, Evolution-300LC, England) and adjusted to 0.5 to obtain a uniform population of bacteria (log 0.95 cfu/ml) bacterial population [54].

The experiment consisted of two factors, i.e., B seed coating and individual and/or combined use of seed inoculation with BTB or biochar application. Four B seed coating levels (0.00, 1.00,1.50, 2.00 g kg^−1^ seed) were included in the study. Boric acid (17 % B) was used as B source. Arabic gum was used as an adhesive material for seed coating. The seeds were first inoculated with bacteria by soaking them in bacteria culture for 2 h and then coated with B. The required amount of B according to doses was mixed with Arabic gum was adhered to the seeds by homogenous shaking for 1 h. Seeds were allowed to dry under shade for 6 h after coating. Pots (50 cm height and 20 cm diameter) were filled with 20 kg soil and biochar (5 % w/w) was applied to the soil before sowing.

Bacteria-inoculated or non-inoculated and B-coated or non-coated seeds (according to treatments) were sown in the pots. Ten seeds were sown in each pot. After complete emergence, six plants (4 for recording growth data at different intervals and two for yield traits) were kept for collecting growth and yield data. One plant was carefully uprooted from each pot during each sampling (i.e., 25, 50, 75 and 100 days after sowing). The pots were irrigated before uprooting the plants on each sampling, which did not damage the roots. Recommended doses of NPK (250:75:75 kg ha^−1^) fertilizers were applied to each pot using ammonium nitrate, triple superphosphate and potassium chloride as sources. The pots were fertilized with 3.38 g ammonium nitrate, 0.72 triple superphosphate and 0.55 g potassium chloride. Nitrogen was applied in three splits, first as a basal dose on sowing. Other doses were applied as top dressing at 35 and 50 days after sowing. Atrazine herbicide was applied as a pre-emergence for weed control. The pots were irrigated when necessary to avoid moisture stress. No insect, pest, and disease infestation were recorded on the crop during the whole growth period; therefore, no plant protection measures were taken. The experiment was laid out according to randomized complete block design with split pot arrangements. Boron seed coating levels were kept in the main plots, whereas seed inoculation and biochar combinations were randomized in sub plots. Each treatment had three replications and consisted of two pots.

### 2.3 Observations

Data relating to different growth parameters, root system, and biochemical and yield-related traits were recorded using the standard procedures described below.

#### 2.3.1 Allometric and root traits

Leaf area (LA), plant growth rate (PGR) and root traits were recorded by uprooting one plant at 25, 50, 75, and 100 days after sowing (DAS) from each pot. The randomly selected plant from each pot at each harvest was carefully uprooted to avoid damage to the roots. Roots were separated from the stem and carefully washed. Root length was measured with the help of measuring tape. Afterwards, the roots were dried at 72 °C for 48 h to record the root growth rate. Root growth rate was measured by using equation (1).

eq. 1 $$\text{RGR}=\frac{W_{2}{-W}_{1}}{t_{2}-t_{1}}$$

In the above equation, RGR = root growth rate (g plant^−1^ day^−1^), W_2_ = root biomass (g) at 2nd harvest, W_1_ = root biomass (g) at 1st harvest, t_2_ = days after sowing at 2nd harvest and t_1_ = days after sowing at first harvest.

The area of the leaves present on the harvested plants at each sampling was measured by using leaf area meter (model MK2 DT Area Meter made by Cambridge UK). Afterwards, the stem and leaves were dried in an electric oven at 72 °C for 48 h to get stem dry weight. The stem and root dry weight were added to get plant biomass, which was then used to compute plant growth rate. Plant growth rate was calculated by using equation (2).

eq. 2 $$\text{PGR}=\frac{W_{2}{-W}_{1}}{t_{2}-t_{1}}$$

In the above equation, PGR = plant growth rate (g plant^−1^ day^−1^), W_2_ = plant biomass at 2nd harvest, W_1_ = plant biomass at 1st harvest, t_2_ = days after sowing at 2nd harvest and t_1_ = days after sowing at first harvest.

#### 2.3.2 Physiological traits

Chlorophyll contents were recorded by using a SPAD-502 chlorophyll meter (Konica Mintola Sensing, Inc., Japan). Photosynthetic rate, stomatal conductance, and transpiration ratio were measured by using infrared gas analyzer (IRGA, LI-6400 Portable photosynthesis System, LI-CORE, USA). Two leaves of each plant directly exposed to sunlight were selected for these measurements. Photosynthetic rate, stomatal conductance, and transpiration ratio were recorded at 2000 μmol m^−2^ s^−1^ PAR (saturating light intensity), 350 μmol mol^−1^ ambient CO_2_ concentration and 31 ± 2 °C leaf temperature. The data on these traits were recorded during 10:00 a.m. and 12:00 p.m. These measurements were made at 60 DAS.

#### 2.3.3 Grain boron and protein content

Grain B content was measured using dry ash method [55] and Azomethine-H colorimetric measurements [56]. Porcelain crucibles containing 1g of dry powdered grains were heated to 550 °C in a muffle furnace. The 10 ml of 0.36 N H_2_SO_4_ solution was applied to the ash. It was heated for 20 min in a steam bath, then chilled for an hour at room temperature. Whatman No.1 filter paper was used to remove the residues. The absorbance of the blank, the filtrate, and the standards was measured at a wavelength of 420 nm using spectrophotometer (Model 6300-VIS). Boron concentration was calculated by drawing a calibration curve from the standard measurements and using equation (3).

eq. 3 $$B\;\left(\mathrm{mg}\;{\mathrm{kg}}^{‐1}\right)=\frac{\text{ppm}B\left(\text{calibration}\;\text{curve}\right)\times V}{Wt}$$

In the above equation, V = volume of total extract (ml), W_t_ = dry weight of the sample (g), and ppm = amount of B obtained after spectrophotometer readings from a calibration curve.

The micro Kjeldahl method [57] was used to determine nitrogen content of each sample. An oven-dried sample was used in the long Kjeldahl flask before crushing. The 5 g catalyst mixture (copper sulfate, ferrous sulfate, and potassium sulfate), and 25 ml of concentrated commercial sulfuric acid (98 %) were added to the crushed samples. A digestion rack was used to heat the samples until the solution was clear. The 250 ml volumetric flask was used to dilute the flask contents with distilled water. The 250 mg of zinc dust and 40 % sodium hydroxide solution were added to a 10 ml aliquot, which was then distilled in the micro Kjeldahl distillation apparatus. Methyl red and boric acid were used to indicate the presence of ammonia in a beaker, which was titrated against ordinary 0.1 N sulfuric acid solutions until the distillate reached a light pink color. The equation (4) was used to determine the nitrogen content:

eq. 4 $$\text{Nitrogen}\;\left(\%\right)=\;\frac{ml\;NH_{2}SO_{4}\times 0.0014\times 250\times 100}{w\times 10}$$

In the equation, W = weight of final sample.

Crude protein was determined by using the equation (5).

eq. 5 Crude protein (%) = Nitrogen (%) × 6.25

Where 6.25 is a standard factor that assumes that protein is composed of 16 % nitrogen.

#### 2.3.4 Yield and related traits

Yield-related traits, i.e., plant height, cob length, number of grains per row, number of grains per cob, number of grain rows, biological yield, grain yield and harvest index were recorded at maturity by using standard procedures. The height of all plants at harvest was measured and averaged for each treatment. The lengths of all cobs in each treatment were measured and averaged to record cob length. Number of grain rows, number of grains per cob and number of grain rows on each cob in each treatment were carefully counted and averaged. The biomass of the harvested plants was measured to record biological yield. The harvested cobs for each treatment were threshed manually and resulting grains were weighed to record grain yield.

### 2.4 Statistical analysis

The collected data (except periodic data for allometric and root traits) were analyzed by analysis of variance (ANOVA). Normality (Shapiro-Wilk normality test) and homogeneity of variance (Levene's test) in the data were tested prior to ANOVA which indicated a normal distribution. Therefore, original data were used in the analysis as normality assumption was fulfilled. Two-way ANOVA was used to infer the significance in the data. Least significant difference (LSD) post-hoc test at 95 % probability was used to compare treatment means where ANOVA denoted significant differences [58]. The statistical software Statistix 8.1 (Florida, USA) was used to analyze the data. The periodic data of allometric and root traits were presented as line graphs prepared in Microsoft Excel (Microsoft Corporation, Washington, United States).

## 3 Results

**Fig. 2:**
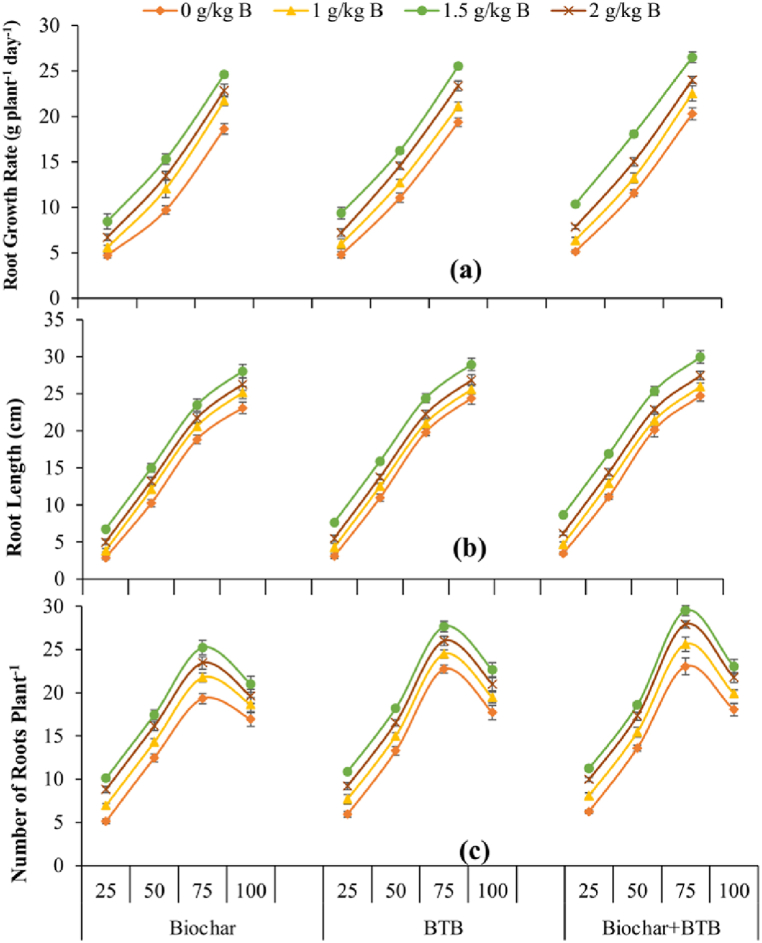
Root growth rate (a), root length (b) and number of roots per plant (c) of maize grown with boron seed coating, seed inoculation with boron tolerant bacteria and biochar application on saline soil. The values presented are means ± standard errors of the means (n = 3). The numbers in the x-axis present days after sowing, whereas text denotes bioinoculants. BTB = boron tolerant bacteria.

Root length and root growth rate continuously increased till 100 days after sowing (DAS), whereas number of lateral roots reached the highest value at 75 DAS and then start declining (Fig. 2a and b). The highest root growth rate, root length and number of lateral roots were recorded for the combined use of B seed coating at 1.5 g kg^−1^ seed and biochar + BTB inoculation at all samplings. The lowest values of these traits were noted for no seed coating + biochar application at all samplings (Fig. 2a, b, 2c).

**Fig. 3:**
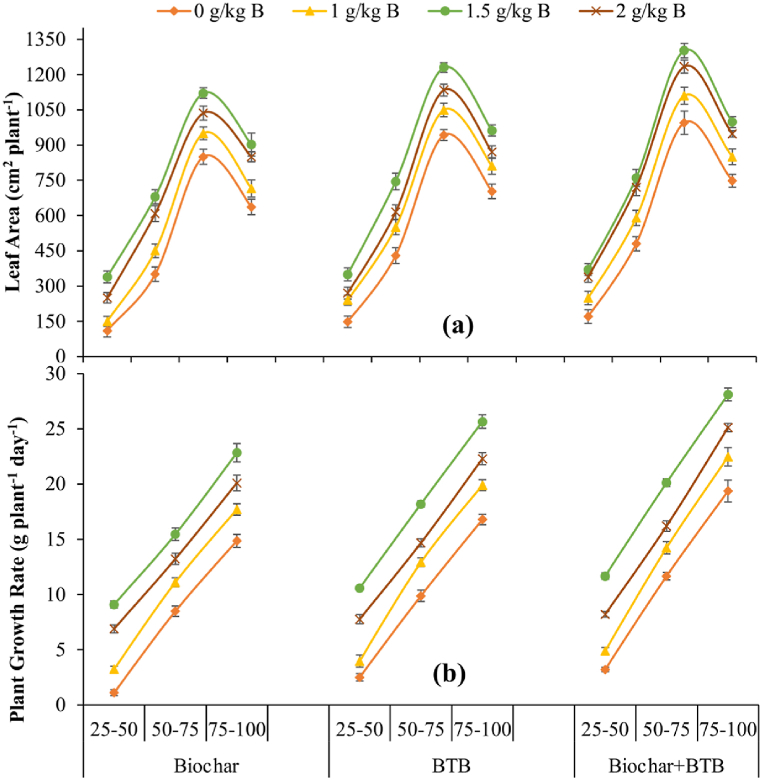
Leaf area (a) and plant growth rate (b) of maize grown with boron seed coating, seed inoculation with boron tolerant bacteria and biochar application on saline soil. The values presented are means ± standard errors of the means (n = 3). The numbers in the x-axis present days after sowing, whereas text denotes bioinoculants. BTB = boron tolerant bacteria.

Leaf area increased until 75 DAS and then started declining, whereas plant growth rate linearly increased till 100 DAS. The interactive effect of B seed coating and biochar + BTB significantly improved leaf area compared to the rest of the combinations included in the study (Fig. 3a and b). The highest leaf area was recorded for the interactive effect of B seed coating at 1.5 g kg^−1^ seed and biochar + BTB, whereas the lowest value was recorded for no seed coating and biochar application (Fig. 3a). Plant growth rate was significantly improved by interactive effect of B seed coating and biochar + BTB inoculation. The interactive effect of B seed coating at 1.5 g kg^−1^ seed and biochar + BTB resulted in the highest plant growth rate, whereas the lowest values were recorded for no seed coating + biochar application at all sampling dates (Fig. 3b).

The individual and interactive effects of B seed coating, and biochar + seed inoculation with BTB significantly affected physiological attributes, yield and related traits and grain quality (Table 1).

**Table 1:** Analysis of variance (p values) for individual and combined effect of B seed coating and biochar + seed inoculaiton wth BTB on physiological, qualitative, and yield-related parameters of maize crop grown on saline soil.

Traits	Boron levels (B)	Biochar + BTB (BI)	B × BI
Chlorophyll content (μmol m^2^)	0.0000**	0.0178*	0.0088**
Stomatal conductance (mmol m^2^ s^1^)	0.0000**	0.0000**	0.0000**
Transpiration Ratio (μmol m^2^ s^1^)	0.0000**	0.0001**	0.0022**
Photosynthetic Rate (mmol m^2^ s^1^)	0.0000**	0.0008**	0.0021**
Boron content (mg kg^−1^)	0.0000**	0.0001**	0.0000**
Protein content (%)	0.0000**	0.0002**	0.0007**
Plant height (cm)	0.0000**	0.0001**	0.0015**
Cob length (cm)	0.0000**	0.0066**	0.4444^NS^
Number of grain rows cob^−1^	0.0000**	0.0177*	0.0023**
Number of grains row^−1^	0.0000**	0.0045**	0.0017**
Number of grains cob^−1^	0.0000**	0.0040**	0.0009**
100-grain weight (g)	0.0000**	0.0001**	0.0000**
Biological yield (g plant^−1^)	0.0000**	0.0000**	0.0000**
Grain yield (g plant^−1^)	0.0000**	0.0001**	0.0001**
Harvest index (%)	0.0000**	0.0803^NS^	0.1506^NS^

* = significant at p 0.05, ** = significant at p = 0.01, NS = non-significant, BTB = boron-tolerant bacteria.

Boron seed coating combined with biochar + seed inoculation with BTB significantly affected yield and yield-related traits. Interactive effect of B seed coating and biochar + seed inoculation with BTB significantly increased plant height, cob length, number of grain rows per cob, number of grains per row, and grains per cob as compared to no seed coating. The highest values for yield-related traits were recorded for the interactive effect of B seed coating at 1.5 g kg^−1^ seed combined with biochar + BTB, whereas combination of no seed coating and biochar application only resulted in the lowest values of these traits. Boron seed coating at 1.5 g kg^−1^ seed combined with biochar + BTB improved plant height by 46 %, cob length by 35 %, number of grain rows cob^−1^ by 26 %, number of grains row^−1^ by 40 %, and number of grains cob^−1^ by 55 % compared to no seed coating combined with biochar application only (Table 2). The highest 100-grain weight (27 g) was recorded with the interactive effect of B seed coating at 1.5 g kg^−1^ seed and biochar + BTB (Table 2).

**Table 2:** Individual and interactive effects of boron seed coating and biochar + seed inoculation with boron tolerant bacteria on growth and yield-related traits of maize crop grown on saline soil.

Treatments	Plant height (cm)				Cob length (cm)			
	Biochar + BTB				Biochar + BTB			
B seed coating	b1	b2	b3	Means	b1	b2	b3	Means
0 (control)	115.0 g	101.3 h	109.3 gh	108.5 D	14.9g	15.6 g	16.3 fg	15.6 D
1 g kg^−1^ seed	124.6 f	135.0 e	146.6 d	135.4C	17.2 ef	17.6 ef	17.1 ef	17.3C
1.5 g kg^−1^ seed	173.0 b	185.6 a	189.0 a	182.5 A	21.6 ab	22.4 a	23.0 a	22.4 A
2 g kg^−1^ seed	154.0 cd	158.0 c	168.0 b	160.0 B	18.0 de	19.1 cd	20.2 bc	19.1B
Means	141.6 B	145.0 B	153.2 A		17.9 B	18.7 A	19.2 A	
LSD (5 %)	B	BI	B × BI		B	BI	B × BI	
	4.46	5.15	8.93		0.73	0.84	1.46	
	Number of grain rows cob^−1^				100-grain weight (g)			
0 (control)	13.5 ef	11.4 g	13.1 f	12.6C	18.5 f	16.1 h	17.2 g	17.3 D
1 g kg^−1^ seed	14.2 de	14.4 cd	14.6 bcd	14.4 B	19.5 f	21.1 e	22.4 d	21.0C
1.5 g kg^−1^ seed	15.0 abc	15.3 ab	15.5 a	15.3 A	26.1 ab	26.8 a	27.0 a	26.6 A
2 g kg^−1^ seed	14.6 bcd	14.7 bcd	14.8 abcd	14.7 B	23.2 d	24.5 c	25.7 b	24.5 B
Means	14.3 A	13.9 B	14.5 A		21.8 B	22.1 B	23.0 A	
LSD (5 %)	B	BI	B × BI		B	BI	B × BI	
	0.38	0.44	0.77		0.49	0.56	0.98	
	Number of grains row^−1^				Number of grains cob^−1^			
0 (control)	16.5 f	14.4 g	15.1 g	15.3 D	223.7 f	165.1 h	197.6 g	195.5 D
1 g kg^−1^ seed	18.4 e	19.6 e	21.2 d	19.8C	263.7 e	283.7 e	311.7 d	286.4C
1.5 g kg^−1^ seed	22.7 bc	23.2 ab	24.3 a	23.4 A	341.3 abc	351.0 ab	364.7 a	352.3 A
2 g kg^−1^ seed	21.8 cd	22.1 bcd	22.6 bc	22.2 B	320.0 cd	327.1 cd	337.0 bc	328.0 B
Means	19.8 B	19.9 B	20.8 A		287.2 B	281.7 B	302.8 A	
LSD (5 %)	B	BI	B × BI		B	BI	B × BI	
	0.61	0.71	1.23		11.94	13.79	23.89	

Here, B = boron seed coating levels, BI = biochar + seed inoculation with boron tolerant bacteria, b1 = biochar, b2 = seed inoculation with boron tolerant bacteria, b3 = biochar + seed inoculation with boron tolerant bacteria, BTB = boron tolerant bacteria. The means followed by different letters within a column or row are statistically different from each other (p ≤ 0.05).

Seed coating at 1.5 g kg^−1^ seed combined with biochar + BTB resulted in the highest increase in grain and biological yields (Table 3). Harvest index was also improved (14 %) with the interactive effect of B seed coating at 1.5 g kg^−1^ seed and biochar + BTB (Table 3).

**Table 3:** The influence of individual and interactive effects of boron seed coating and biochar + seed inoculation with boron tolerant bacteria on grain and biological yields, and harvest index of maize crop grown on saline soil.

Treatments	Grain yield (g plant^−1^)				Biological yield (g plant^−1^)			
	Biochar + BTB				Biochar + BTB			
B seed coating	b1	b2	b3	Means	b1	b2	b3	Means
0 (control)	38.4 g	33.0 i	35.9 h	35.8 D	74.7 h	67.3 j	71.4 i	71.1 D
1 g kg^−1^ seed	40.6 f	42.3 f	44.5 e	42.5C	77.5 g	80.3 f	83.5 e	80.4C
1.5 g kg^−1^ seed	52.1 b	53.1 ab	54.9 a	53.4 A	92.3 bc	93.9 ab	95.5 a	93.9 A
2 g kg^−1^ seed	46.0 de	47.8 d	50.1 c	48.0 B	85.3 e	87.8 d	90.6 c	87.9 B
Means	44.3 B	44.1 B	46.4 A		82.4 B	82.3 B	85.2A	
LSD (5 %)	B	BI	B × BI		B	BI	B × BI	
	0.95	1.10	1.91		0.92	1.06	1.85	
	Harvest index (%)							
0 (control)	51.6 fg	49.2 h	50.4 gh	50.4 D				
1 g kg^−1^ seed	52.5 ef	52.7 ef	53.3 de	52.9C				
1.5 g kg^−1^ seed	56.5 b	56.6 ab	57.6 a	56.9 A				
2 g kg^−1^ seed	54.08 cde	54.5 cd	55.3 bc	54.6 B				
Means	53.62 AB	53.2 B	54.2 A					
LSD (5 %)	B	BI	B × BI					
	0.79	0.91	1.58					

Here, B = boron seed coating levels, BI = biochar + seed inoculation with boron tolerant bacteria, b1 = biochar, b2 = seed inoculation with boron tolerant bacteria, b3 = biochar + seed inoculation with boron tolerant bacteria, BTB = boron tolerant bacteria. The means followed by different letters within a column or row are statistically different from each other (p ≤ 0.05).

Photosynthetic rate, transpiration ratio, and stomatal conductance were significantly improved with interactive effect of B seed coating and biochar + BTB. Boron seed coating at 1.5 g kg^−1^ seed combined with biochar + BTB improved photosynthetic rate by 15 %, transpiration ratio by 52 %, and stomatal conductance by 27 % as compared to B seed coating at 0 g kg^−1^ seed combined with biochar only (Table 4). Similarly, chlorophyll contents were improved by 33 % with B seed coating at 1.5 g kg^−1^ seed combined with biochar + BTB compared to no seed coating combined with biochar only (Table 4).

**Table 4:** Individual and interactive effects of boron seed coating and biochar + boron tolerant bacteria on physiological and qualitative parameters of maize grown on saline soil.

Treatments	Stomatal conductance (mmol m^−2^ s^−1^)				Photosynthetic rate (mmol m^−2^ s^−1^)			
	Biochar + BTB				Biochar + BTB			
B seed coating	b1	b2	b3	Means	b1	b2	b3	Means
0 (control)	105.7 h	98.2 i	103.7 h	102.6 D	34.9 g h	33.3 i	34.3 h	34.1 D
1 g kg^−1^ of seed	109.9 g	111.3 g	114.3 f	111.8C	35.3f g	35.5 fg	36.1 ef	35.6C
1.5 g kg^−1^ of seed	129.3 c	132.9 b	135.7 a	132.6 A	37.4 bc	38.2 b	39.4 a	38.4 A
2 g kg^−1^ of seed	118.5 e	121.6 d	127.3 c	122.5 B	36.4 de	36.7 cde	37.2 cd	36.8 B
Means	115.9 B	116.0 B	120.2 A		36.0 B	35.9 B	36.7 A	
LSD (5 %)	B	BI	B × BI		B	BI	B × BI	
	1.38	1.59	2.76		0.41	0.47	0.82	
	Transpiration ratio (μmol m^−2^ s^−1^)				Chlorophyll content (μmol m^−2^)			
0 (control)	8.2 g	6.6 h	7.8 g	7.5 D	28.7 fg	25.2 h	27.1 gh	27.0 D
1 g kg^−1^ of seed	9.1 f	9.6 f	10.5 e	9.7C	29.5 f	31.5 e	32.1 de	31.1C
1.5 g kg^−1^ of seed	12.9 b	13.2 ab	13.8 a	13.3 A	35.1 bc	35.8 b	37.8 a	36.2 A
2 g kg^−1^ of seed	11.0 de	11.4 d	12.1 c	11.5 B	33.2 cde	33.6 cd	34.4 bc	33.7 B
Means	10.3 B	10.2 B	11.0 A		31.6 B	31.5 B	32.8 A	
LSD (5 %)	B	BI	B × BI		B	BI	B × BI	
	0.34	0.40	0.69		0.96	1.11	1.93	
	Protein content (%)				Boron content (mg kg^−1^)			
0 (control)	8.3 g	6.4 i	7.5 h	7.4 D	63.4 h	57.2 j	61.2 i	60.6 D
1 g kg^−1^ of seed	9.1 f	9.6 f	10.5 e	9.7C	65.6 g	67.6 f	69.6 e	67.6C
1.5 g kg^−1^ of seed	12.9 b	13.2 ab	13.9 a	13.3 A	72.8 ab	73.6 a	74.1 a	73.5 A
2 g kg^−1^ of seed	11.0 de	11.4 d	12.1 c	11.5 B	70.3 de	71.3 cd	72.0 bc	71.2 B
Means	10.3 B	10.1 B	11.0 A		68.0 B	67.4 B	69.2 A	
LSD (5 %)	B	BI	B × BI		B	BI	B × BI	
	0.36	0.42	0.73		0.68	0.78	1.36	

Here, B = boron seed coating levels, BI = biochar + boron tolerant bacteria, b1 = biochar, b2 = seed inoculation with boron tolerant bacteria, b3 = biochar + seed inoculation with boron tolerant bacteria, BTB = boron tolerant bacteria. The means followed by different letters within a column or row are statistically different from each other (p ≤ 0.05).

Boron seed coating combined with biochar + BTB significantly improved grain protein and B content. Boron seed coating at 1.5 g kg^−1^ seed combined with biochar + BTB improved grain B content by 22 % and grain protein content by 53 % as compared to no seed coating combined with biochar only (Table 4).

## 4 Discussion

Salinity hindered the uptake of essential nutrients, which resulted in reduced leaf area, photosynthesis, biomass accumulation, and yield of maize in the current study. Poor root growth, reduced leaf area and lower yield were recorded in the current study under salinity. It is well known that salinity impedes plant growth, resulting in reduced biomass accumulation [7]. The decreased crop yield and productivity under saline conditions can be attributed to stunted root growth and reduced leaf size [8]. Elevated salt concentration exerts detrimental effects on moisture availability, which lead to decreased yield [6] and similar findings were recorded in the current study.

Boron seed coating combined with biochar + seed inoculation with BTB (*Bacillus* sp. MN-54) significantly improved physiological and yield-related traits of maize grown under saline environments as hypothesized. Seed inoculation with BTB combined with B seed coating and biochar application also improved root growth in the current study [6] compared to the combination of no seed coating and biochar only. Better root growth improved soil organic matter, exchangeable potassium, and available phosphorus. Biochar application improved soil physical properties, which resulted in increased root growth [59]. Free et al. [60] reported increased root growth of maize with biochar application. Similarly, Zahir et al. [61] recorded that seed inoculation with bacteria significantly improved root growth of maize. Increased chlorophyll contents improved leaf area under B seed coating combined with biochar and seed inoculation with BTB in the current study. Similar results have been reported by Gaurav [62] who found that B application significantly increased chlorophyll and relative water contents of maize, which resulted in improved leaf area. Similarly, biochar application increased the availability of primary nutrients, i.e., phosphorous, potassium, calcium, and magnesium, in soil. The enhanced nutrient availability can be owed to increased nutrient retention, elevated cation exchange capacity, soil pH buffering, promotion of microbial activity, improvement of water holding capacity, and reduction of nutrient losses. Furthermore, possible improvements in physical, chemical, and biological properties of soil might resulted in better plant growth under salinity [[36], [37], [38], [39]].

Bacteria significantly improve plant growth; therefore, regarded as plant growth-promoting rhizobacteria. These bacteria colonize plant roots and lead to improved nutrient solubilization, nitrogen fixation, production of plant growth regulators, and stress tolerance [63]. Our finding indicated that combined effect of biochar, B seed coating and BTB inoculation improved leaf area and photosynthesis rate. Higher salt concentration alters structure of microbial communities [64]. Biochar application been found to enhance soil aggregate formation and potentially enhance the growth and development of soil microorganisms [65]. The addition of biochar has been reported to improve the density of bacteria belonging to *Bacillus* genus, which improve plant growth [66]. Better growth and yield-related attributes in the current study in the combined application of biochar + BTB are owed to improved soil properties and bacteria density.

Chlorophyll content, photosynthetic rate, transpiration ratio, and stomatal conductance were improved by the interactive effect of B seed coating and biochar + seed inoculation with BTB. These results are comparable to Muhammad et al. [51] who reported that combined application of *Bacillus* sp. MN-54 and biochar resulted in a considerable increase in photosynthetic rate and the transpiration ratio. Improved nitrogen uptake due to B seed coating, bacteria inoculation and biochar application might be responsible for improvement in leaf chlorophyll contents [67].

Grain protein and B contents significantly increased with B seed coating combined with biochar + seed inoculation with BTB. The findings of Bonilla et al. [68] support the results of the increase in grain protein contents caused by B application. Improved B concentration may be attribute to the genetic availability of a large pool of B inside the plant tissues [69]. However, seed coating of B combined with biochar and BTB play a role in reserve mobilization may be the possible explanations for greater nutrient sequestration in grains.

Boron seed coating and biochar + BTB significantly increased plant height. Kaya et al. [70] reported that B application increased plant height. Better root growth due to B application combined with biochar + seed inoculation with BTB improved yield-related traits (100-grain weight, grain rows per cob, cob length, and grains per cob). The results of Butnan et al. [71] are similar to the results of this study who reported that exogenous B application enhanced cob length and the number of grains per cob in hybrid maize.

Boron plays an important impact in enhancing cob diameter [72]. The findings of the current study indicated that B application significantly improved different yield-related parameters of maize. Rahim et al. [73] also observed an increased number of grains per cob in maize plants with B application. Several studies showed that B application improved biological and grain yield in different crops [74,75]. This increase in maize grain yield can be attributed to improved nutrient-use efficiency, higher photosynthetic rates, and integrated reserve translocation [76]. The accelerated synthesis of carbohydrates and their transport to grains is another factor contributing to the increased yield that can be attributed to the application of micronutrients [77]. Better nutrient uptake and seedling development significantly improved growth-related attributes, ultimately resulting in higher biological and grain yields [78].

## 5 Conclusions

Boron seed coating at 1.5 g kg^−1^ seed integrated with biochar and seed inoculation with *Bacillus* sp. MN-54 significantly improved photosynthesis, transpiration, chlorophyll contents, yield-related traits, and grain quality of maize grown on saline soil. Therefore, this combination can be used to improve maize growth and grain quality on saline soil. However, field studies with more biochar concentrations and types are needed for more sound results and large-scale recommendations. Furthermore, the mechanisms underlying the improved growth and yield should be investigated in the future studies.

## Funding

This work was funded by the Researchers Supporting Project number (RSPD2023R686), 10.13039/501100002383King Saud University, Riyadh, Saudi Arabia.

## Data availability statement

The data used in the manuscript has not been used in any repository and all data are provided within the manuscript.

## CRediT authorship contribution statement

**Hafiz Saqib Hayat:** Data curation, Software, Validation, Visualization, Writing – original draft. **Atique-ur Rehman:** Conceptualization, Methodology, Supervision, Validation, Writing – review & editing. **Shahid Farooq:** Conceptualization, Validation, Visualization, Writing – review & editing. **Muhammad Naveed:** Conceptualization, Methodology, Resources, Writing – review & editing. **Hayssam M. Ali:** Funding acquisition, Methodology, Resources, Writing – review & editing. **Mubshar Hussain:** Conceptualization, Supervision, Writing – review & editing.

## Declaration of competing interest

The authors declare that they have no known competing financial interests or personal relationships that could have appeared to influence the work reported in this paper.
